# Polymorphism, expression and structure analysis of key genes in the ovarian steroidogenesis pathway in sheep (*Ovis aries*)

**DOI:** 10.1002/vms3.485

**Published:** 2021-03-29

**Authors:** Wen‐Ping Hu, Ming‐Qiu Liu, Zhi‐Long Tian, Qiu‐Yue Liu, Zhuang‐Biao Zhang, Ji‐Shun Tang, Xiao‐Yun He, Yan‐Yan Zhu, Yuan‐Yuan Wang, Ming‐Xing Chu

**Affiliations:** ^1^ Key Laboratory of Animal Genetics and Breeding and Reproduction of Ministry of Agriculture and Rural Affairs Institute of Animal Sciences Chinese Academy of Agricultural Sciences Beijing China; ^2^ Department of Biology Science Bengbu Medical College Bengbu China; ^3^ Institute of Genetics and Developmental Biology The Innovation Academy for Seed Design Chinese Academy of Sciences Beijing China; ^4^ Institute of Animal Husbandry and Veterinary Medicine Anhui Academy of Agricultural Sciences Hefei China

**Keywords:** *CYP11A1*, *HSD3B1*, litter size, ovarian steroidogenesis pathway, sheep, *STAR*

## Abstract

**Background:**

Litter size is an important factor that significantly affects the development of the sheep industry. Our previous TMT proteomics analysis found that three key proteins in the ovarian steroidogenesis pathway, STAR, HSD3B1, and CYP11A1, may affect the litter size trait of Small Tail Han sheep.

**Objective:**

The purpose of this study was to better understand the relationship between polymorphisms of these three genes and litter size.

**Material and Method:**

Sequenom MassARRAY detected genetic variance of the three genes in 768 sheep. Real‐time qPCR of the three genes was used to compare their expression in monotocous and polytocous sheep in relevant tissues. Finally, bioinformatics analysis predicted the protein sequences of the different SNP variants.

**Result:**

Association analysis showed that there was a significant difference in litter size among the genotypes at two loci of the *CYP11A1* gene (*p* < 0.05), but no significant difference was observed in litter size among all genotypes at all loci of the *STAR* and *HSD3B1* genes (*p* > 0.05). However, *STAR* expression was significantly different in polytocous and monotocous sheep in the pituitary (*p* < 0.01). Tissue‐specific expression in the ovary was observed for *HSD3B1* (*p* < 0.05), but its expression was not different between polytocous and monotocous sheep. Bioinformatics analysis showed that the g.33217408C > T mutation of *CYP11A1* resulted in major changes to the secondary and tertiary structures. In contrast, gene polymorphisms in *STAR* and *HSD3B1* had minimal impacts on their protein structures.

**Discussion:**

This may explain why the CYP11A1 variant impacted litter size while the others did not. The single nucleotide polymorphism of the *CYP11A1* gene would serve as a good molecular marker when breeding to increase litter size in sheep. Our study provides a basis for further revealing the function of the ovarian steroidogenesis pathway in sheep reproduction and sheep breeding.

## Graphical abstract

It was speculated that the single nucleotide polymorphism of the *CYP11A1* gene was a potential molecular marker to increase the litter size of sheep. It provided a basis for further revealing the function of ovarian steroidogenesis pathway in sheep reproduction and sheep breeding.

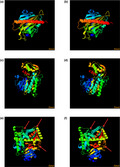

## 1 INTRODUCTION

Since the domestication of sheep, their various products have become important staples in society, including their meat, wool, skin, milk and others. Among them, mutton is an important source of protein for humans. Mutton sheep production plays an important role in the animal husbandry economy. The sheep industry prioritizes three traits to maximize economic benefits: reproduction, meat production and fur. Of these, improved reproductive traits yield the most gains in revenue. The economic benefit of double lambing is more than 1.6 times that of single lambing (Notter, 2012). There are 700 sheep breeds in the world; the 71 in China are comprised of 42 local breeds, eight introduced breeds and 21 cultivated breeds. Only a few breeds consistently birth more than one sheep at a time; these so‐called polytocous sheep have a lambing rate of more than 200%. Statistically, most species of sheep are seasonal oestrus and monotocous—generally yielding only one lamb per litter per year. There are some famous sheep breeds with a lambing rate of 200% or above, including Booroola Merino, Inverdale, Cambridge, Thoka, Javanese, Olkuska, Belclare, Lacaune and Woodlands sheep, as well as Hu sheep and Small Tail Han sheep in China (Zheng et al., 2019). The Booroola Merino average lambing rate can reach 350%, while the average lambing rate of Small Tail Han sheep and Hu sheep are above 267.1% and 277.4%, respectively. Despite their lower lambing rate, these Chinese varieties have more consistent performance in lamb production, a result of long‐term natural selection and artificial selection (National Committee on Livestock and Poultry Genetic Resources, 2012). Reproduction is extremely complicated; environmental (Greives et al., 2007), nutritional (Abecia et al., 2015) and genetic factors (McLaren, 1998) all play an important role in the regulation of sheep reproductive processes.

In 1982, Davis et al. identified the main gene controlling litter size in Booroola Merino sheep in Australia (Davis et al., 1982). In 1989, it was officially named FecB (Fec = fecundity, B = Booroola). Since then, the search for other major genes has become the goal of sheep geneticists. Small Tail Han is a famous polytocous sheep breed from China (Tang et al., 2019). This breed has three different FecB genotypes (BB, B+ and ++). In our previous TMT proteomics study, the ovarian steroidogenesis pathway was significantly enriched (according to KEGG pathway analysis) in the ovaries of Small Tail Han sheep lacking the FecB mutation (FecB++). It led us to hypothesize that the key proteins of the ovarian steroidogenesis pathway, STAR (steroidogenic acute regulatory protein), HSD3B1 (hydroxy‐delta‐5‐steroid dehydrogenase, 3 beta‐ and steroid delta‐isomerase 1) and CYP11A1 (Cytochrome P450 family 11 subfamily A member 1), affect litter size (Tang et al., 2019).

Steroid hormones control metabolism, inflammation, immune functions, salt and water balance, development of sexual characteristics, and the ability to withstand illness and injury (Busada & Cidlowski, 2017; Frye, 2009; Fujita, 2010). In terms of pharmacological action, there are three types of steroid hormones, corticosteroid, sex hormone and mineralocorticoid. Sex hormones are further subdivided into male and female hormones. They are related to sex and the development of secondary sexual characteristics in animals. Oestrogen is found in all vertebrates and is responsible for the maturation and maintenance of the vagina and uterus. It is also involved in ovarian functions, such as the maturation of ovarian follicles. Oestrogen also plays an important role in the regulation of gonadotropin secretion (Baker, 2013; Nelson & Bulun, 2001). Progestogens are another type of steroid hormones that are secreted by luteal cells in the ovary. Their main physiological functions support a safe pregnancy: they inhibit ovulation, promote endometrium secretion, facilitate fertilized egg implantation and reduce uterine muscle excitement (Haas & Ramsey, 2013; Wahabi et al., 2018). Thus, steroid hormones are necessary for successful reproduction in females.

The ovarian steroidogenesis pathway is closely related to ovarian function and follicular development (Gareis et al., 2018). This pathway includes *STAR*, *HSD3B1*, *CYP1A1*, *CYP1B1*, *CYP11A1*, *CYP17A1* and *CYP19A1*. These genes encode the enzymes that synthesize steroids in the ovary. Steroidogenic acute regulatory protein, encoded by the *STAR* gene, mediates the transfer of cholesterol from the outer mitochondrial membrane to the inner mitochondrial membrane, where lysis to gestenolone occurs. Thus, STAR indirectly increases the progesterone levels in the body that affect ovulation and reproductive functions (Di Renzo et al., 2016; Hanukoglu et al., 1977; Kohen et al., 2003; Tajima et al., 2003; Vallee, 2016). *HSD3B1* encodes 3β‐hydroxyl steroid dehydrogenase/isomerase (3β‐HSD), which plays a crucial role in the biosynthesis of all hormonal steroids. This enzyme affects ovulation by converting pregnenolone to progesterone (Koritz, 1964). The proteins encoded by *CYP1A1*, *CYP1B1*, *CYP11A1*, *CYP17A1* and *CYP19A1* are monooxygenases in the cytochrome p450 superfamily. In sheep and other mammals, these proteins are important for the removal of xenobiotics, as well as the synthesis and breakdown of endogenous hormones, including oxidized steroids, and fatty acids (Danielson, 2002; Gonzalez & Gelboin, 1992). Cytochrome P450 side chain lyase (P450scc) encoded by the *CYP11A1* gene is a mitochondrial enzyme that plays an important role in the regulation of the catalytic conversion of cholesterol to progesterone (O'Hara et al., 2014).

The *STAR*, *HSD3B1* and *CYP11A1* genes of the ovarian steroidogenesis pathway were directly involved in the synthesis of the steroid hormone, progesterone. Our previous quantitative mass spectrometry data revealed an association between these proteins and the polytocous trait. But there were no relevant studies on the correlation between *STAR*, *HSD3B1* and *CYP11A1* genes with the litter size of sheep. To better understand the effects of these three genes, Sequenom MassARRAY technology was used to detect gene polymorphisms in seven sheep breeds. The association between polymorphism of these genes and litter size was analysed. The expression levels of these genes were also measured in Small Tail Han ewes, in order to understand the relationship between gene expression and litter size. Our findings could provide a reference for further research on molecular marker‐assisted breeding of sheep.

## 2 MATERIALS AND METHODS

### 2.1 Blood sample collection and DNA extraction

Jugular blood samples (10‐ml blood per sheep) from 768 sheep of seven breeds were collected into EDTA‐coated tubes. Genomic DNA was extracted using blood genomic DNA extraction kits (TIANGEN Biotech Co., Ltd.) and stored at −20°C. The isolated DNA was measured by agarose gel electrophoresis to ensure its quality.

Small Tail Han sheep (*n* = 384), Cele Black sheep (*n* = 68), and Hu sheep (*n* = 83) were the polytocous sheep breeds in this study. Tan sheep (*n* = 23), Prairie Tibetan (*n* = 80), Suffolk sheep (*n* = 60) and Sunite sheep (*n* = 70) were the monotocous sheep breeds in this study. The selected sheep were of similar age and fed in an indoor setting under similar conditions of room temperature, illumination, feeding system and nutrition level.

### 2.2 Genotyping

According to the early re‐sequencing data (version 4.1) from our laboratory consisting of 100 individual sheep from 10 different breeds (Pan et al., 2018), SNP loci of the *STAR, HSD3B1* and *CYP11A1* genes were screened. The g.31959747T>C, g.31962677G>A, g.31959875C>A and g.31960614C>T loci in the *STAR* gene; g.96094239C>T, g.96095151G>A, g.96101288G>A, g.96092576G>A and g.96101542C>A loci in the *HSD3B1* gene; and the g.33222725A>G and g.33217408C>T loci in the *CYP11A1* gene were selected for genotyping in 768 polytocous and monotocous sheep, including Small Tail Han sheep, Cele Black sheep, Hu sheep, Tan sheep, Prairie Tibetan sheep, Suffolk sheep and Sunite sheep using MassARRAY (Zhang et al., 2019). First, the invariant region of each gene was amplified. This initial amplification was then subjected to single‐base extended PCR using specific primers. The molecular weight of products after extension, which differs due to incorporation of different bases at polymorphic sites, were analysed by time‐of‐flight mass spectrometry to identify the different genotypes (Zhang et al., 2019).

### 2.3 Statistical analysis

Allele frequencies, genotype frequencies, *p* values, polymorphism information content (*PIC*), heterozygosity (*HE*) and the number of effective alleles (*NE*) were calculated using the data obtained from genotyping results. Sheep populations with *p* > 0.05 (chi‐square test) were considered to conform to the Hardy–Weinberg equilibrium. A linear model using the equation $y_{\mathrm{ijn}}=\mu +P_{i}+G_{j}+I_{\text{PG}}+e_{\mathrm{ijn}}$ was subsequently applied to analyse the association of genotypes and litter size, in which *y_ijn_* represents the phenotypic value (litter size); *μ* is the population mean; P*_i_* is the fixed effect of the i*th* parity (*i* = 1, 2, or 3); G*_j_* represents the effect of the j*th* genotypes (*j* = 1, 2, or 3); I_PG_ represents the interactive effect of parity and genotype; and e*_ijn_* represents random error.

### 2.4 Gene expression in the reproductive axis of sheep

Small Tail Han sheep were selected randomly from the polytocous and monotocous groups. The tissue samples of this experiment were collected from a farm at the Tianjin Animal Husbandry and Veterinary Research Institute. The RNA was extracted from the hypothalamus, pituitary and ovary tissues according to the instructions of the RNA extraction kits (TIANGEN Biotech Co., Ltd.). Primer Premier 6.0 and Beacon Designer 8.0 were used for to design primers for *STAR*, *HSD3B1* and *CYP11A1*, and the internal reference gene glyceraldehyde 3‐phosphate dehydrogenase (*GAPDH*) using sequences from GenBank.

Reverse transcription PCR (RT‐PCR) was performed using the PrimeScript^™^ RT reagent Kit. The 20‐µl reaction solution contained: 1‐µl PrimeScript RT Enzyme Mix I, 1‐µl Oligo dT Primer, 1‐µl Random 6 mers, 4‐µl 5X PrimeScript Buffer (for Real‐Time), 1‐µg RNA, and RNase‐free ddH_2_O. The reaction conditions of RT‐PCR were: 37°C for 15 min and 85°C for 5 s.

The 20‐µl quantitative real‐time PCR (RT‐qPCR) reaction system contained: 10.0‐µl SYBR Premix Ex Taq II, 0.8‐µl upstream and downstream primers, 2.0‐µl cDNA template, and 6.4‐µl RNase‐free ddH_2_O. The PCR procedure started with pre‐denaturation at 95°C for 5 s, followed by 40 cycles of denaturation at 95°C for 5 s and denaturation at 60°C for 30 s. To determine relative expression, the CT value of each gene in Small Tail Han sheep hypothalamic, pituitary and ovary tissues was obtained by RT‐qPCR. Next, the ΔCT was calculated as CT value–average CT value of *GAPDH*, then the ΔΔCT value was calculated as the ΔCT value–average ΔCT value of *GAPDH*. Finally, the relative expression was represented as 2^−ΔΔCT^.

### 2.5 Bioinformatics analysis

First, the sequences of the genes were obtained from NCBI (https://www.ncbi.nlm.nih.gov/), and the amino acid sequences were obtained from UniProt (https://www.uniprot.org/). Prediction of the secondary structure of each gene and its variants was carried out using PredictProtein (https://www.predictprotein.org/). The three‐dimensional (3‐D) structures before and after mutation were predicted with Phyre2 (Kelley et al., 2015). The transmembrane domains before and after mutation were predicted using TMHMM (Krogh et al., 2001).

## 3 RESULTS

### 3.1 Population genetic analysis of known SNPs in STAR, HSD3B1 and CYP11A1 genes

The SNPs identified in *STAR*, *HSD3B1*, and *CYP11A1* genes are listed in Table 1. Each of the three possible genotypes at the g.31959747T>C and g.31962677G>A loci of the *STAR* gene (Table 2), and at the g.96094239C>T and g.96101542C>A loci of the *HSD3B1* gene was detected in the population of tested sheep (Table 3). However, only two genotypes were detected at the SNP loci of *CYP11A1* (Table 4). The data showed that only the g.31962677G>A locus of the *STAR* gene and the g.96094239C>T locus of the *HSD3B1* gene was moderately polymorphic (0.25 < *PIC* < 0.5) in all sheep breeds. The g.31959875C>A locus of the *STAR* gene was moderately polymorphic (0.25 < *PIC* < 0.5) in Prairie Tibetan sheep and Suffolk sheep. The g.96101542C>A locus of the *HSD3B1* gene was moderately polymorphic (0.25 < *PIC* < 0.5) in Small Tail Han sheep, Cele Black sheep and Hu sheep. The other loci all had a low‐degree polymorphism (*PIC* < 0.25). According to the available data, the g.31959747T>C locus was not in Hardy–Weinberg equilibrium (*p* < 0.05) in Cele Black sheep, Hu sheep, Tan sheep, Suffolk sheep and Sunite sheep; the g.31959875T>C locus was not in Hardy–Weinberg equilibrium (*p* < 0.05) in Prairie Tibetan sheep and Suffolk sheep; and the g.96101542T>C locus was not in Hardy–Weinberg equilibrium (*p* < 0.05) in Suffolk sheep. All other populations were in Hardy–Weinberg equilibrium (*p* > 0.05).

**TABLE 1:** SNPs observed in *STAR*, *HSD3B*1 and *CYP11A1* genes

Gene	SNP position	Consequence type	Nucleotide change	Codon change	Amino acid change
*STAR*	g.31959747	3′ UTR variant	T>C	—	—
	g.31962677	missense variant	G>A	GCC/GTC	A/V
	g.31959875	3′ UTR variant	C>A	—	—
	g.31960614	missense variant	C>T	CGC/CAC	R/H
*HSD3B1*	g.96094239	intron variant	C>T	—	—
	g.96095151	downstream variant	G>A	—	—
	g.96101288	missense variant	G>A	CGG/CAG	R/Q
	g.96092576	missense variant	G>A	GCC/ACC	A/T
	g.96101542	missense variant	C>A	CTG/ATG	L/M
*CYP11A1*	g.33222725	synonymous variant	A>G	TCA/TCG	—
	g.33217408	missense variant	C>T	TCC/TTC	S/F

**TABLE 2:** Polymorphism of *STAR* gene and population genetic analysis in seven sheep breeds

	Breed	Number	Genotype frequency			Allele frequency		*PIC*	*HE*	*NE*	Chi‐square test (*p* value)
			CC	CT	TT	C	T				
g.31959747T>C	Small Tail Han	378	0.01 (2)	0.02 (8)	0.97 (368)	0.02	0.98	0.03	0.03	1.03	0.73
	Cele Black	68	0.01 (1)	0.01 (1)	0.98 (66)	0.02	0.98	0.03	0.03	1.03	0.00
	Hu	83	0.06 (5)	0.08 (7)	0.86 (71)	0.10	0.90	0.16	0.18	1.22	0.00
	Tan	23	0.04 (1)	0.00 (0)	0.96 (22)	0.04	0.96	0.07	0.08	1.08	0.00
	Prairie Tibetan	80	0.03 (2)	0.16 (13)	0.81 (65)	0.11	0.89	0.18	0.20	1.24	0.20
	Suffolk	60	0.02 (1)	0.02 (1)	0.96 (58)	0.03	0.97	0.06	0.06	1.06	0.00
	Sunite	70	0.09 (6)	0.04 (3)	0.87 (61)	0.11	0.89	0.18	0.20	1.24	0.00
			AA	AG	GG	A	G				
g.31962677G>A	Small Tail Han	384	0.12 (48)	0.49 (188)	0.39 (148)	0.37	0.63	0.36	0.47	1.89	0.32
	Cele Black	68	0.12 (8)	0.50 (34)	0.38 (26)	0.37	0.63	0.36	0.47	1.87	0.53
	Hu	83	0.10 (8)	0.48 (40)	0.42 (35)	0.34	0.66	0.35	0.45	1.81	0.47
	Tan	23	0.13 (3)	0.48 (11)	0.39 (9)	0.37	0.63	0.36	0.47	1.87	0.90
	Prairie Tibetan	80	0.15 (12)	0.40 (32)	0.45 (36)	0.35	0.65	0.35	0.46	1.83	0.27
	Suffolk	60	0.10 (6)	0.40 (24)	0.50 (30)	0.30	0.70	0.33	0.42	1.72	0.71
	Sunite	70	0.20 (14)	0.41 (29)	0.39 (27)	0.41	0.60	0.37	0.48	1.93	0.24
			CC	CA	AA	C	A				
g.31959875C>A	Small Tail Han	289	1.00 (289)	0.00 (0)	0.00 (0)	1.00	0.00	0.00	0.00	1.00	—
	Cele Black	60	0.88 (53)	0.12 (7)	0.00 (0)	0.94	0.06	0.11	0.11	1.13	0.63
	Hu	78	0.79 (62)	0.21 (16)	0.00 (0)	0.90	0.11	0.17	0.19	1.23	0.31
	Tan	21	0.81 (17)	0.19 (4)	0.00 (0)	0.91	0.10	0.16	0.17	1.21	0.63
	Prairie Tibetan	64	0.52 (33)	0.48 (31)	0.00 (0)	0.76	0.24	0.30	0.36	1.57	0.01
	Suffolk	50	0.48 (24)	0.52 (26)	0.00 (0)	0.74	0.26	0.31	0.38	1.63	0.01
	Sunite	65	0.72 (47)	0.28 (18)	0.00 (0)	0.86	0.14	0.21	0.24	1.32	0.20
			CC	CT	TT	C	T				
g.31960614C>T	Small Tail Han	384	0.99 (380)	0.01 (4)	0.00 (0)	0.99	0.01	0.02	0.02	1.02	0.92
	Cele Black	68	0.97 (66)	0.03 (2)	0.00 (0)	0.99	0.02	0.03	0.03	1.03	0.90
	Hu	83	1.00 (83)	0.00 (0)	0.00 (0)	1.00	0.00	0.00	0.00	1.00	—
	Tan	23	1.00 (23)	0.00 (0)	0.00 (0)	1.00	0.00	0.00	0.00	1.00	—
	Prairie Tibetan	80	0.96 (77)	0.04 (3)	0.00 (0)	0.98	0.02	0.04	0.04	1.04	0.86
	Suffolk	60	0.98 (59)	0.02 (1)	0.00 (0)	0.99	0.01	0.02	0.02	1.02	0.95
	Sunite	70	1.00 (70)	0.00 (0)	0.00 (0)	1.00	0.00	0.00	0.00	1.00	—

Abbreviations: HE, heterozygosity; *NE*, number of effective alleles; *PIC*, polymorphism information content.

**TABLE 3:** Polymorphism of the *HSD3B1* gene and population genetic analysis in seven sheep breeds

	Breed	Number	Genotype frequency			Allele frequency		*PIC*	*HE*	*NE*	Chi‐square test (*p* value)
			CC	CT	TT	C	T				
g.96094239C>T	Small Tail Han	383	0.11 (44)	0.41 (157)	0.48 (182)	0.32	0.68	0.34	0.44	1.77	0.26
	Cele Black	68	0.16 (11)	0.59 (40)	0.25 (17)	0.46	0.55	0.37	0.50	1.98	0.13
	Hu	82	0.15 (12)	0.45 (37)	0.40 (33)	0.38	0.63	0.36	0.47	1.88	0.76
	Tan	23	0.09 (2)	0.57 (13)	0.34 (8)	0.38	0.63	0.36	0.47	1.88	0.31
	Prairie Tibetan	80	0.06 (5)	0.35 (28)	0.59 (47)	0.24	0.77	0.29	0.36	1.56	0.76
	Suffolk	60	0.03 (2)	0.43 (26)	0.54 (32)	0.25	0.76	0.30	0.37	1.59	0.23
	Sunite	70	0.09 (6)	0.33 (23)	0.58 (41)	0.26	0.75	0.31	0.38	1.61	0.30
			GG	GA	AA	G	A				
g.96095151G>A	Small Tail Han	383	1.00 (383)	0.00 (0)	0.00 (0)	1.00	0.00	0.00	0.00	1.00	—
	Cele Black	68	1.00 (68)	0.00 (0)	0.00 (0)	1.00	0.00	0.00	0.00	1.00	—
	Hu	83	0.98 (81)	0.02 (2)	0.00 (0)	0.99	0.01	0.02	0.02	1.02	0.91
	Tan	22	1.00 (22)	0.00 (0)	0.00 (0)	1.00	0.00	0.00	0.00	1.00	—
	Prairie Tibetan	80	1.00 (80)	0.00 (0)	0.00 (0)	1.00	0.00	0.00	0.00	1.00	—
	Suffolk	60	1.00 (60)	0.00 (0)	0.00 (0)	1.00	0.00	0.00	0.00	1.00	—
	Sunite	70	1.00 (70)	0.00 (0)	0.00 (0)	1.00	0.00	0.00	0.00	1.00	—
			GG	GA	AA	G	A				
g.96101288G>A	Small Tail Han	380	0.99 (377)	0.01 (3)	0.00 (0)	0.99	0.01	0.02	0.02	1.02	0.94
	Cele Black	68	1.00 (68)	0.00 (0)	0.00 (0)	1.00	0.00	0.00	0.00	1.00	—
	Hu	82	1.00 (82)	0.00 (0)	0.00 (0)	1.00	0.00	0.00	0.00	1.00	—
	Tan	23	1.00 (23)	0.00 (0)	0.00 (0)	1.00	0.00	0.00	0.00	1.00	—
	Prairie Tibetan	80	1.00 (80)	0.00 (0)	0.00 (0)	1.00	0.00	0.00	0.00	1.00	—
	Suffolk	60	1.00 (60)	0.00 (0)	0.00 (0)	1.00	0.00	0.00	0.00	1.00	—
	Sunite	70	1.00 (70)	0.00 (0)	0.00 (0)	1.00	0.00	0.00	0.00	1.00	—
			GG	GA	AA	G	A				
g.96092576G>A	Small Tail Han	383	0.68 (262)	0.3 (114)	0.02 (7)	0.83	0.17	0.24	0.28	1.39	0.14
	Cele Black	68	1.00 (68)	0.00 (0)	0.00 (0)	1.00	0.00	0.00	0.00	1.00	—
	Hu	83	1.00 (83)	0.00 (0)	0.00 (0)	1.00	0.00	0.00	0.00	1.00	—
	Tan	23	1.00 (23)	0.00 (0)	0.00 (0)	1.00	0.00	0.00	0.00	1.00	—
	Prairie Tibetan	80	1.00 (80)	0.00 (0)	0.00 (0)	1.00	0.00	0.00	0.00	1.00	—
	Suffolk	60	1.00 (60)	0.00 (0)	0.00 (0)	1.00	0.00	0.00	0.00	1.00	—
	Sunite	70	1.00 (70)	0.00 (0)	0.00 (0)	1.00	0.00	0.00	0.00	1.00	—
			CC	CA	AA	C	A				
g.96101542G>A	Small Tail Han	382	0.65 (248)	0.32 (121)	0.03 (13)	0.81	0.19	0.26	0.31	1.44	0.71
	Cele Black	68	0.47 (32)	0.44 (30)	0.09 (6)	0.69	0.31	0.34	0.43	1.75	0.78
	Hu	83	0.63 (52)	0.33 (27)	0.04 (4)	0.80	0.21	0.27	0.33	1.48	0.84
	Tan	23	0.78 (18)	0.17 (4)	0.05 (1)	0.87	0.14	0.21	0.23	1.30	0.26
	Prairie Tibetan	80	0.85 (68)	0.13 (10)	0.02 (2)	0.92	0.09	0.14	0.16	1.18	0.05
	Suffolk	49	0.93 (45)	0.05 (3)	0.02 (1)	0.96	0.04	0.08	0.09	1.09	0.00
	Sunite	70	0.69 (48)	0.29 (20)	0.02 (2)	0.84	0.17	0.24	0.28	1.38	0.96

Abbreviations: HE, heterozygosity; *NE*, number of effective alleles; *PIC*, polymorphism information content.

**TABLE 4:** Polymorphism of *CYP11A1* gene and population genetic analysis in seven sheep breeds

	Breed	Number	Genotype frequency			Allele frequency		*PIC*	*HE*	*NE*	Chi‐square test (*p* value)
			AA	AG	GG	A	G				
g.33222725A>G	Small Tail Han	383	0.97 (370)	0.03 (13)	0.00 (0)	0.99	0.01	0.03	0.03	1.03	0.74
	Cele Black	68	0.96 (65)	0.04 (3)	0.00 (0)	0.98	0.02	0.04	0.04	1.04	0.85
	Hu	85	0.96 (82)	0.04 (3)	0.00 (0)	0.98	0.02	0.04	0.04	1.04	0.87
	Tan	23	0.87 (20)	0.13 (3)	0.00 (0)	0.94	0.06	0.11	0.12	1.14	0.74
	Prairie Tibetan	80	0.81 (65)	0.1 9(15)	0.00 (0)	0.91	0.10	0.16	0.17	1.21	0.35
	Suffolk	60	0.97 (58)	0.03 (2)	0.00 (0)	0.99	0.02	0.03	0.03	1.03	0.90
	Sunite	70	0.90 (63)	0.10 (7)	0.00 (0)	0.95	0.05	0.09	0.10	1.10	0.66
			CC	CT	TT	C	T				
g.33217408C>T	Small Tail Han	384	0.98 (377)	0.02 (7)	0.00 (0)	0.99	0.01	0.02	0.02	1.02	0.85
	Cele Black	68	0.96 (65)	0.04 (3)	0.00 (0)	0.98	0.02	0.04	0.04	1.04	0.85
	Hu	83	0.98 (81)	0.02 (2)	0.00 (0)	0.99	0.01	0.02	0.02	1.02	0.91
	Tan	23	0.96 (22)	0.04 (1)	0.00 (0)	0.98	0.02	0.04	0.04	1.04	0.92
	Prairie Tibetan	80	0.96 (77)	0.04 (3)	0.00 (0)	0.98	0.02	0.04	0.04	1.04	0.86
	Suffolk	60	0.98 (59)	0.02 (1)	0.00 (0)	0.99	0.01	0.02	0.02	1.02	0.95
	Sunite	70	0.99 (69)	0.01 (1)	0.00 (0)	1.00	0.01	0.01	0.01	1.01	0.95

Abbreviations: HE, heterozygosity; *NE*, number of effective alleles; *PIC*, polymorphism information content.

### 3.2 Correlation analysis between SNPs and litter size of Small Tail Han sheep

In order to demonstrate the correlation between SNPs and litter size of sheep more intuitively, we carried out association analysis between the number of individuals of each genotype and litter size (Table 5). The result of litter size is expressed as the average with standard deviation. No significant difference in litter size between different genotypes of both the *STAR* and *HSD3B1* genes was found (*p* > 0.05). However, there was a significant difference in litter size between different genotypes of the *CYP11A1* gene (*p* < 0.05). The g.33222725A>G locus was associated with litter size at all parities, and sheep with the AA genotype had a greater litter size than those with the AG genotype (*p* < 0.05). The g.33217408C>T SNP also correlated with litter size at all parities, and sheep with the CT genotype had a greater litter size than those with CC genotype (*p* < 0.05).

**TABLE 5:** Association of different SNP genotypes of the *STAR*, *HSD3B1* and *CYP11A1* genes with litter size in Small Tail Han Sheep

Gene	Locus	Genotype	First parity litter size (*N*)	Second parity litter size (*N*)	Third parity litter size (*N*)
*STAR*	g.31959747T>C	TT	1.86 ± 0.03 *N* = 368	2.11 (220) ± 0.05	2.41 (85) ± 0.09
		CT	2.00 ± 0.27 (8)	2.33 (3) ± 0.33	3.50 (2) ± 0.50
		CC	1.50 (2) ± 0.50	1.00 (1) ± 0.00	0.00 (0) ± 0.00
	g.31962677G>A	GG	1.91 (148) ± 0.06	2.10 (88) ± 0.09	2.35 (31) ± 0.14
		GA	1.85 (188) ± 0.05	2.12 (117) ± 0.07	2.48 (48) ± 0.14
		AA	1.85 (48) ± 0.10	2.13 (24) ± 0.15	2.50 (10) ± 0.16
	g.31959875C>A	CC	1.89(289) ± 0.04	2.15 (176) ± 0.06	2.43 (67) ± 0.10
	g.31960614C>T	CC	1.87 (380) ± 0.03	2.11 (226) ± 0.05	2.45 (87) ± 0.09
		CT	2.50 (4) ± 0.29	2.67 (3) ± 0.33	2.00 (2) ± 1.00
*HSD3B1*	g.96094239C>T	CC	1.89 (44) ± 0.10	2.30 (27) ± 0.13	2.46 (13) ± 0.27
		CT	1.89 (157) ± 0.05	2.08 (97) ± 0.08	2.59 (39) ± 0.14
		TT	1.85 (182) ± 0.05	2.09 (104) ± 0.08	2.27 (37) ± 0.12
	g.96095151G>A	GG	1.87 (383) ± 0.03	2.11 (228) ± 0.05	2.44 (88) ± 0.09
	g.96101288G>A	GG	1.87 (377) ± 0.03	2.11 (226) ± 0.05	2.43 (88) ± 0.09
		GA	1.67 (3) ± 0.33	1.00 (1) ± 0.00	0.00 (0) ± 0.00
	g.96092576G>A	GG	1.87 (381) ± 0.03	2.11 (228) ± 0.05	2.43 (88) ± 0.09
	g.96101542C>A	CC	1.88 (248) ± 0.04	2.15 (149) ± 0.06	2.55 (64) ± 0.11
		CA	1.87 (121) ± 0.06	2.06 (67) ± 0.09	2.15 (20) ± 0.15
		AA	1.69 (13) ± 0.17	1.91 (11) ± 0.25	2.00 (4) ± 0.41
*CYP11A1*	g.33222725A>G	AA	1.88 (370) ± 0.04^a^	2.22 (218) ± 0.05^a^	2.47 (87) ± 0.09^a^
		AG	1.68 (13) ± 0.18^b^	2.00 (10) ± 0.24^b^	2.00 (4) ± 0.42^b^
	g.33217408C>T	CC	1.88 (377) ± 0.03^b^	2.10 (227) ± 0.05^b^	2.42 (89) ± 0.09^b^
		CT	2.00 (7) ± 0.25^a^	2.50 (2) ± 0.53^a^	2.90 (2) ± 0.57^a^

Note

Different letters for the groups indicate differences (*p* < 0.05).

### 3.3 Gene expression in the reproductive axis of sheep

**FIGURE 1:**
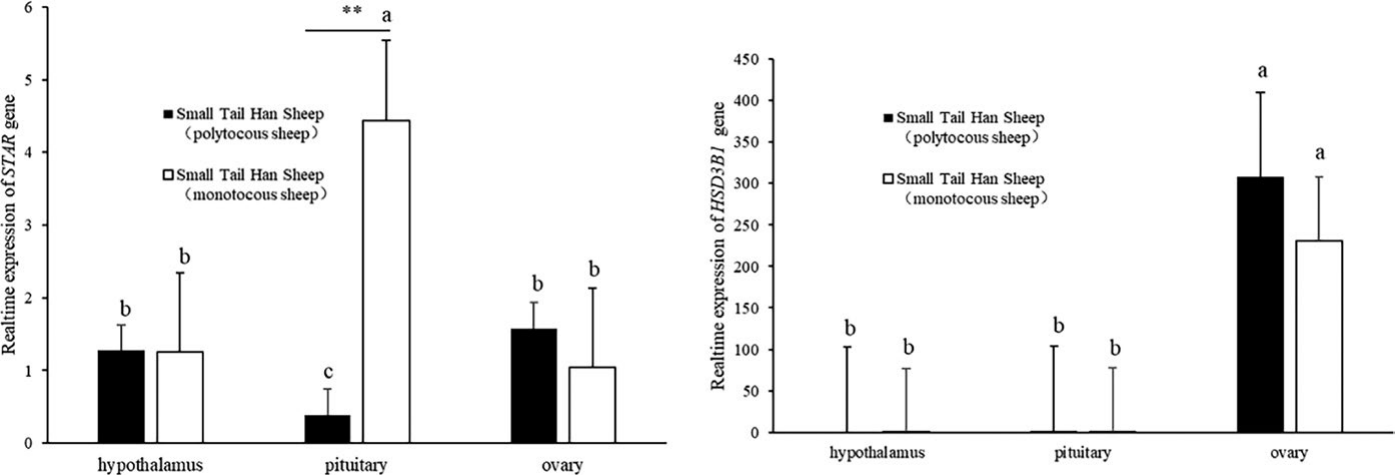
Real time‐qPCR analysis of *STAR* and *HSD3B1* expression in hypothalamus, pituitary and ovary tissues of Small Tail Han sheep. Different letters denote a significant difference (*p* < 0.05), and ** corresponds to *p* < 0.01

Gene expression in hypothalamic, pituitary and ovary tissues of Small Tail Han sheep was studied between monotocous and polytocous populations by RT‐qPCR (Figure 1). The *STAR* gene was expressed in all three tissues, and there was a significant difference between polytocous sheep and monotocous sheep in the pituitary (*p* < 0.01). No significant difference between polytocous sheep and monotocous sheep of the *HSD3B1* gene in the ovary was found (*p* > 0.05). But the *HSD3B1* gene was mainly expressed in ovary, and not in the other two tissues (*p* < 0.05). According to our previous study (Tian et al., 2019), the *CYP11A1* gene is also mainly expressed in ovary, and its expression in monotocous sheep is significantly lower than that in polytocous sheep (*p* < 0.05).

### 3.4 Bioinformatics analysis

Several SNPs resulted in amino acid changes (Table 1). In the STAR gene, SNPs g.31962677G>A and g.31960614C>T resulted in amino acid changes at residue 103 (Ala to Val) and residue 274 (Arg to His), respectively. In *HSD3B1*, SNPs g.96101288 G>A, g.96092576 G>A and g.96101542 C>A caused amino acid changes at residue 285 (Arg to Gln), residue 2 (Ala to Thr) and residue 370 (Leu to Met. SNP g.33217408C>T of *CYP11A1* caused an amino acid change at residue 41 (Ser to Phe).

**FIGURE 2:**
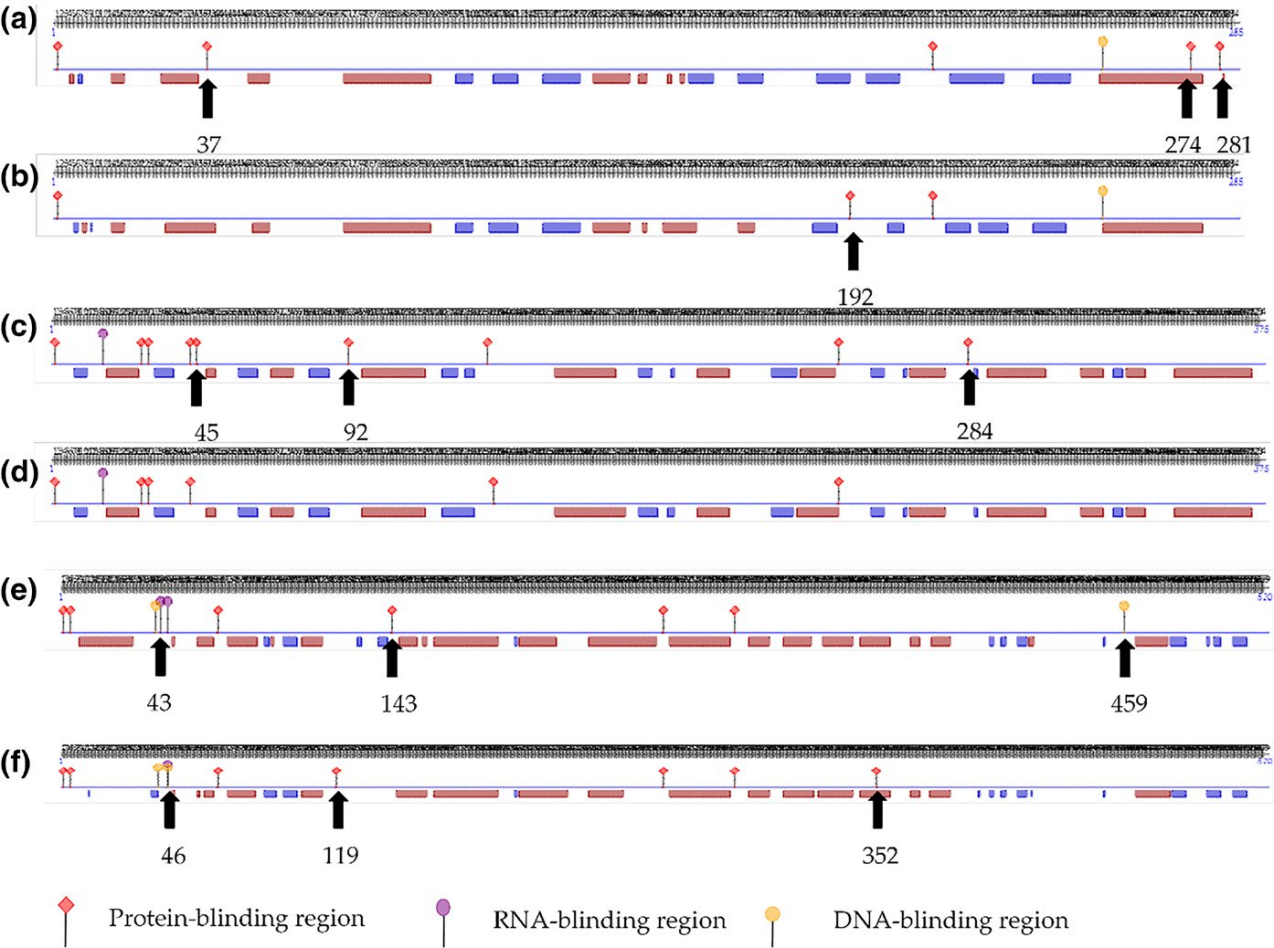
Protein secondary structure of STAR, HSD3B1 and CYP11A1 before and after mutations caused by multiple SNPs simultaneously. Predicted secondary protein structure of STAR before mutation (a) and after the g.31962677G>A and g.31960614C>T mutations (b); Secondary protein structure of HSD3B1 before mutation (c) and after the g.96101288G>A, g.96092576G>A and g.96101542C>A mutations (d); and secondary protein structure of CYP11A1 before mutation (e) and after the g.33217408C>T mutation (f)

The effects of these amino acid changes on the protein secondary structure was predicted by PredictProtein (Kelley et al., 2015). For the STAR protein, the three protein‐binding regions in proximity to amino acids 37, 274 and 281 (Figure 2a) were no longer present after simultaneous mutations at g.31962677G>A and g.31960614C>T, and there was a new protein‐binding region in proximity to amino acid 192 (Figure 2b). HSD3B1 had three protein‐binding regions near amino acid numbers 45, 92 and 284 (Figure 2c), but after mutations at g.96101288G>A, g.96092576G>A and g.96101542C>A, they were absent (Figure 2d). For CYP11A1, there was an RNA‐binding region in proximity to amino acid number 43, one protein‐binding region near amino acid 143, and one DNA‐binding region near amino acid 459 (Figure 2e); these binding sites were no longer present after the g.33217408C>T mutation, but two new protein‐binding regions in proximity to amino acids 119 and 352, and one DNA‐binding region in proximity to amino acid 46 were present after the g.33217408C>T mutation (Figure 2f). Unlike STAR and HSD3B1 proteins, CYP11A1 had only one SNP mutation. However, the changes in the secondary structure of CYP11A1 protein were more complex, which may be one reason why the gene was significantly associated with litter size.

**FIGURE 3:**
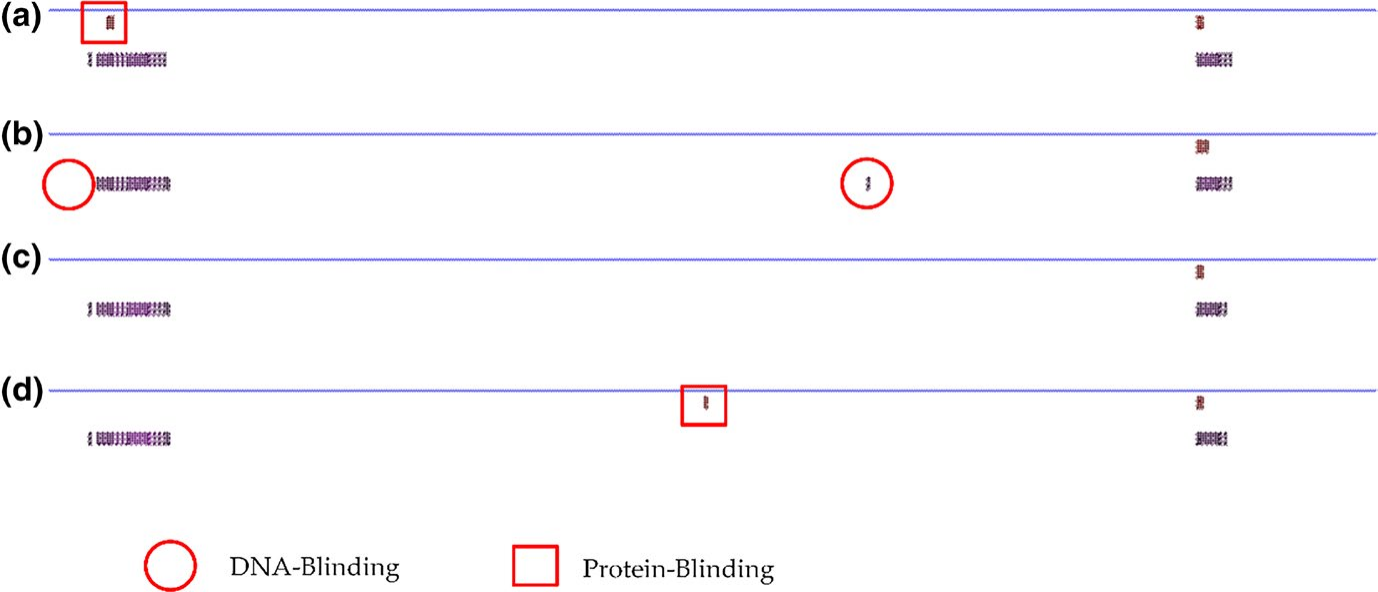
Predicted changes in secondary structure of the STAR protein before and after one or two SNP mutations. Secondary protein structure before mutation (a); after the g.31962677G>A mutation (b); after the g.31960614C>T mutation (c); and after the g.31962677G>A and g.31960614C>T mutations (d)

**FIGURE 4:**
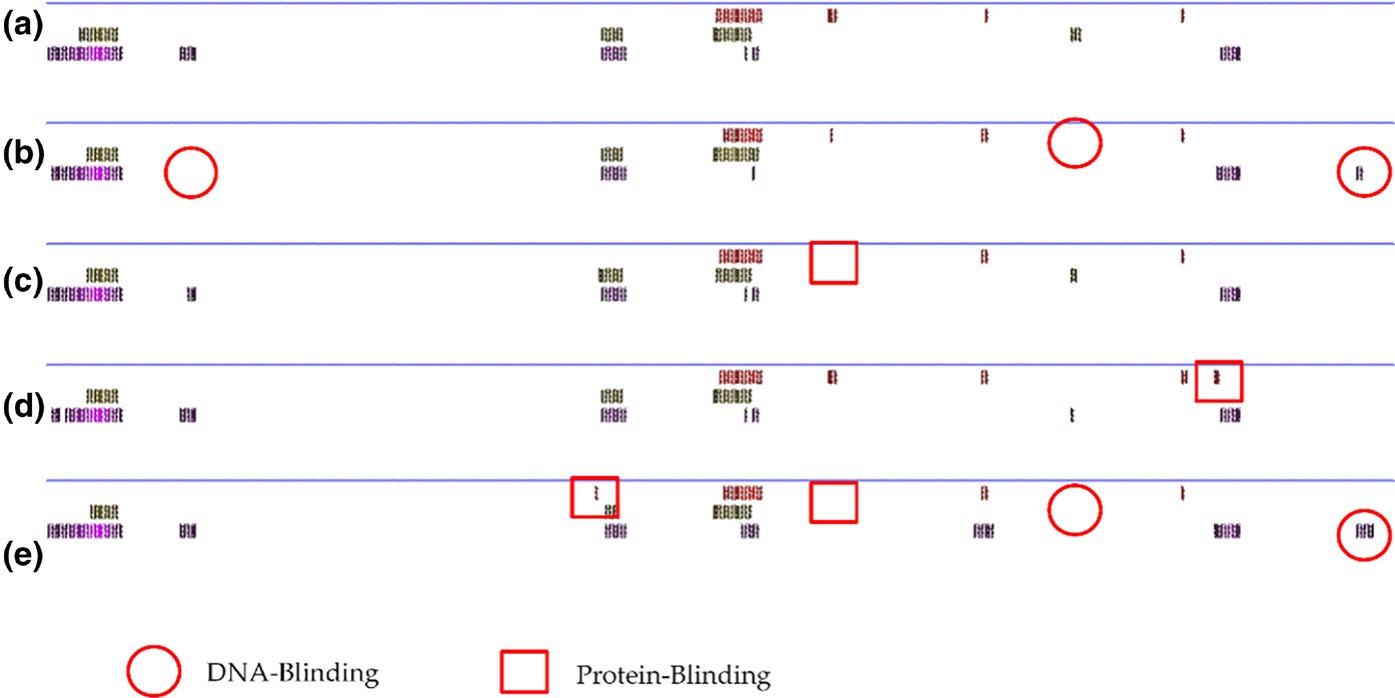
Predicted changes in secondary structure of the HSD3B1 protein before and after one or more SNP mutations. Secondary protein structure before mutation (a); after the g.96101288G>A mutation (b); after the g.96092576G>A mutation (c); after the g.96101542C>A mutation (d); and after the g.96101288G>A, g.96092576G>A and g.96101542C>A mutations (e)

However, the probability of two or three SNP mutations occurring at the same time is very low, so we analysed the effect of single SNP mutations on protein secondary structure. The results showed that STAR has few protein binding sites to begin with (Figure 3a), and after a single (Figure 3b,c) or multiple SNPs mutations (Figure 3d), there were still few binding sites. *HSD3B1* originally had many binding sites (Figure 4a), but the degree of change caused by a single SNP mutation was low (Figure 4b–d). Only when three SNPs mutated simultaneously was the degree of change was high (Figure 4e); however, the probability of this occurring is extremely low. In comparison, one SNP in *the CYP11A1* gene brings significantly more changes to protein secondary structure (Figure 2e,f), which may explain why this g.33217408C>T SNP was associated with the litter size of sheep. And it might also be the reason why the SNPs in *STAR* and *HSD3B1* gene werje not associated with litter size, even though they also caused amino acid changes.

**FIGURE 5:**
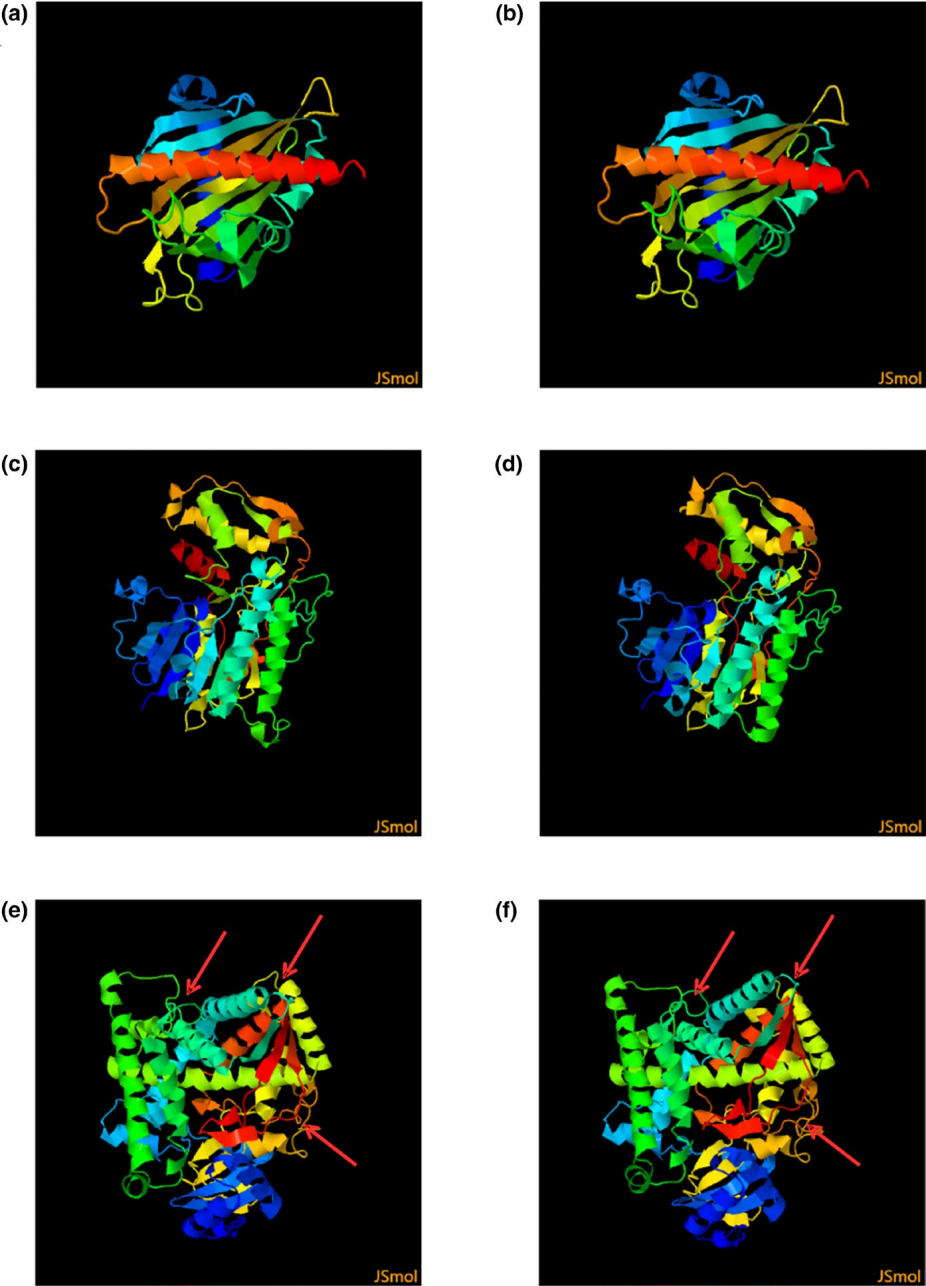
The predicted 3‐D structures of STAR, HSD3B1 and CYP11A1 protein variants before and after the changes caused by SNPs. The predicted 3‐D structure of STAR before (a) and after the mutation caused by the g.31960614C>T SNP (b); the predicted 3‐D structure of HSD3B1 before (c) and after mutation due to the g.96101288G>A SNP (d); and the predicted 3‐D structure of CYP11A1 before (e) and after mutation from g.33217408C>T (f)

The 3‐D structures before and after mutation in STAR, HSD3B1 and CYP11A1 were predicted via Phyre2 (Figure 5). The tertiary structure of CYP11A1 changed significantly after mutation (Figure 5e vs. Figure 5f), whereas STAR and HSD3B1 showed no significant changes. Due to the high similarity of the images, only one mutation result was presented for STAR and HSD3B1 in Figure 5. These 3‐D computational models further confirm that the low degree of variation after single SNP mutation in the secondary structure of STAR and HSD3B1 would not likely affect protein function.

**FIGURE 6:**
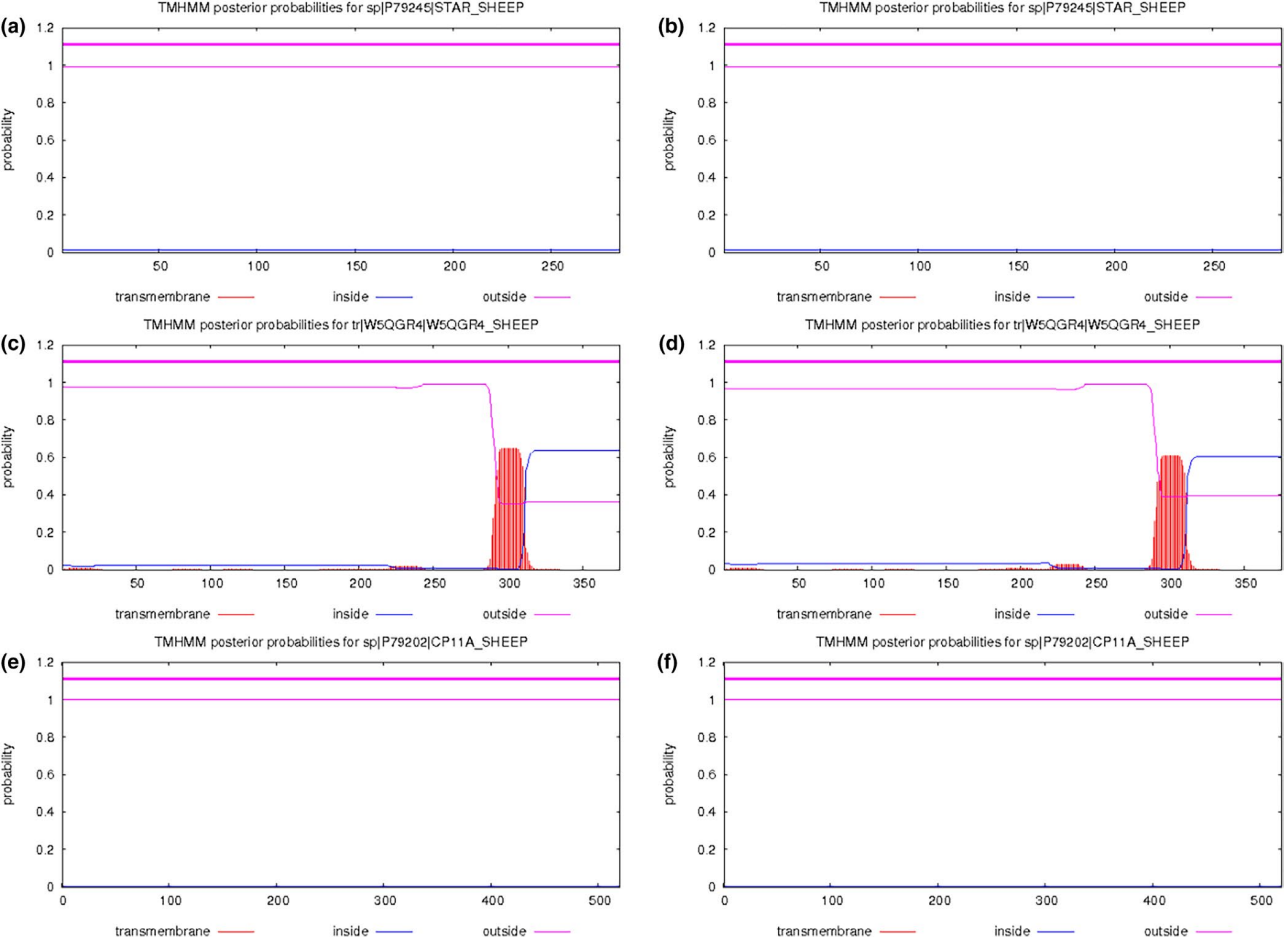
Predicted transmembrane regions of STAR, HSD3B1 and CYP11A1 from Small Tail Han sheep. Predicted transmembrane regions before (a) and after the g.31962677G>A and g.31960614C>T mutations of STAR (b); predicted transmembrane regions before (c) and after the g.96101288G>A, g.96092576G>A and g.96101542C>A mutations of HSD3B1 (d); Predicted transmembrane regions before (e) and after the g.33217408C>T mutations of CYP11A1 (f)

In addition, TMHMM analysis online (Krogh et al., 2001) predicted that the proteins encoded by *STAR* and *CYP11A1* were outside the membrane before and after mutation. HSD3B1 had a predicted transmembrane region around amino acid 300 before and after the mutation. The results suggest that the mutations may not significantly affect the proteins' transmembrane structures (Figure 6).

## 4 DISCUSSION

Progestogens are important steroid hormones in human reproduction. The main tissues affected by progestogens include the uterus, vagina, cervix, breast, testicles and brain. The main effect of progesterone in the body is on the female and the male reproductive systems (Oettel & Mukhopadhyay, 2004). It is important to note that progestogens are often mistakenly confused with progesterone (P4). In fact, P4 is one of the most important progestogens. There are still a variety of different progestogens in the human body, but most of them are metabolites of P4 (Edwards et al., 2005) and are located downstream of P4 from the perspective of biosynthesis. P4 is produced from cholesterol with pregnenolone (P5) as a metabolic intermediate. In all mammals, the ovary is the primary site of P4 production, and the luteal cells of the ovary have key enzymes that convert cholesterol into P5 (via P4). The placenta accounts for the majority of P4 production in sheep, horse, and human (Malassine, 2001), while in other species, the corpus luteum is a major source of P4. Thus, P4 is the primary placental progesterone in sheep, horse and human.

As one of the most important progestogens, P4 is involved in the menstrual cycle, pregnancy and embryogenesis in human and other species (Taraborrelli, 2015). Recent research suggests that P4 affects reproduction (and cancer) through agonistic interactions with membrane P4 receptors (mPRs) (Thomas & Pang, 2012). However, more studies are needed to confirm this route (Valadez‐Cosmes et al., 2016). It is well established that P4 transitions the endometrium into a secretory phase that prepares the uterus for implantation. P4 has an anti‐mitogenic effect in endometrial epithelial cells, which reduces the oestrogenic effect (Patel et al., 2015), and seems to reduce the mother's immune response during implantation and pregnancy, making pregnancy acceptable to the mother's immune system (Di Renzo et al., 2016). Near the time of delivery, P4 reduces the contractile force of uterine smooth muscles (Wahabi et al., 2018), which helps prevent premature delivery (Di Renzo et al., 2016). According to Zakar and Hertelendy (2007), reducing P4 levels may facilitate onset of childbirth.

The ovarian steroidogenesis pathway includes *STAR*, *HSD3B1*, *CYP1A1*, *CYP1B1*, *CYP11A1*, *CYP17A,* and *CYP19A1*. STAR and HSD3B1 are related to the synthesis of sterol hormones (E2, P4 and androgen) that play a crucial role in the normal physiological effects of the ovary and maturation of the follicle (Guo et al., 2018). HSD3B1 and CYP11A1 (P450scc) play important regulatory roles in steroid hormone biosynthesis.

*STAR* is expressed in the ovaries of a variety of animals, mainly overcoming the rate‐limiting transfer of cholesterol from the outer mitochondrial membrane to P450scc (encoded by *CYP11A1*) located in the cell membrane (Yivgi‐Ohana et al., 2009). Polytocous and monotocous Small Tail Han sheep had a similar level of *STAR* expression in ovary (Figure 1). However, *STAR* was highly expressed in the pituitary gland of monotocous sheep and low in the pituitary gland of polytocous sheep (Figure 1). Relevant studies have reported that ACTH (adrenocorticotropic hormone) produced by the anterior pituitary stimulates the adrenal cortex to synthesize glucocorticoids (Clark, 2016). The first step in glucocorticoid synthesis is the transfer of cholesterol to the mitochondrial matrix, which indirectly leads to a dramatic increase in the expression and function of steroid acute regulatory protein (STAR). This explains the high expression of *STAR* in the pituitary of monotocous sheep. However, whether the low expression of *STAR* in the pituitary is related to litter size has not been proved, and further experimental verification is still needed.

HSD3B1, the rate‐limiting enzyme for sex hormone synthesis catalyses the initial step of steroid hormone production. Progesterone secretion by luteal cells requires P450scc and HSD3B1 enzyme activity (Chien et al., 2013). Studies have shown that the highest activity of HSD in the mitochondria of luteal cells may play an important role in the synthesis of progesterone (Chapman et al., 1992). This is consistent with the main expression of *HSD3B1* gene in ovary observed in our study (Figure 1).

CYP19A1 and CYP1A1 in the ovarian steroidogenesis pathway are involved in the formation of ovarian steroids and may have important effects on the maintenance of luteal function and follicular dynamic development. CYP19A1 synthesizes oestrogen from androgen the membrane of granulosa cells into (Miller & Auchus, 2011), thereby promoting follicular development. In vitro, oocytes with low expression of CYP1A1 (which encodes P450scc) had decreased rates of maturation (Pocar et al., 2004). P450scc is a mitochondrial enzyme that regulates the conversion of cholesterol into gestenolone (O'Hara et al., 2014). Also, studies on poultry have confirmed that the *CYP11A1* gene has significant changes in expression during follicular development (Johnson & Woods, 2009), and *CYP11A1* is regulated by hormones during follicular changes (Sechman et al., 2011).

To date, there have only been a few studies analysing SNPs in *CYP11A1*. A minor allele of *CYP11A1*, rs11638442, was proposed to increase endogenous levels of ouabain, an important factor in the cardiovascular system (Tripodi et al., 2009). Besides, six tagging SNPs were examined in the Han Chinese Women Study to prove an association between *CYP11A1* and breast cancer (Sun et al., 2012). Related studies indicated that the SNP‐derived mutations of *CYP11A1* were closely related to reproduction and to the susceptibility to polycystic ovary syndrome, which can cause either chronic ovulation or anovulation (i.e., lack of ovulation) (Zhang et al., 2012). In the previous studies, the expression of the *CYP11A1* gene in the ovarian tissue of monotocous Small Tail Han sheep was significantly higher than that of polytocous sheep (*p* < 0.01), suggesting that the *CYP11A1* gene plays a negative regulation role during oestrus or ovulation (Tian et al., 2019). The *CYP11A1* gene is also expressed in the hypothalamus and pituitary, but the specific role in these tissues still needs to be studied. In this study, the number of *CYP11A1* mutation sites and amino acid changes was less than those of *STAR* and *HSD3B1*. However, according to bioinformatics analysis, the secondary structure change of CYP11A1 protein was more complex than that of STAR and HSD3B1 proteins, and there was a significant difference in litter size before and after mutation (*p* < 0.05). We speculate that g.33222725A>G and g.33217408C>T of *CYP11A1* gene are the key influential mutation sites. Our study suggests that the *CYP11A1* gene may be a polytocous gene; however, further investigation is required.

## 5 CONCLUSIONS

In general, the expression results showed that *STAR* gene was expressed in the hypothalamus‐pituitary‐ovary gonad axis, and there was a significant difference between polytocous sheep and monotocous sheep in the pituitary (*p* < 0.01). The *HSD3B1* gene was mainly expressed in ovary, and there was significant difference between the ovary and other two tissues (*p* < 0.05). There was a significant difference in the association of litter size among the genotypes at g.33222725A>G and g.33217408C>T loci of *CYP11A1* gene (*p* < 0.05). SNP g.33217408C>T of *CYP11A1* caused an amino acid change at residue 41 (Ser to Phe). The physicochemical properties of Ser and Phe differ significantly, and this appeared to result in notable changes to the secondary and tertiary structures of CYP11A1. Our study provides a basis for further revealing the roles of the ovarian steroidogenesis pathway in sheep reproduction.

## CONFLICT OF INTEREST

The authors declare no conflict of interest.

## AUTHOR CONTRIBUTION

**Wenping Hu:** Conceptualization; Data curation; Formal analysis; Funding acquisition; Investigation; Methodology; Project administration; Supervision; Validation; Visualization; Writing‐original draft; Writing‐review & editing. **Mingqiu Liu:** Data curation; Formal analysis; Investigation; Methodology; Software; Validation; Visualization; Writing‐original draft; Writing‐review & editing. **Zhilong Tian:** Data curation; Investigation; Methodology; Validation. **Qiuyue Liu:** Funding acquisition; Resources. **Zhuangbiao Zhang:** Methodology; Software. **Jishun Tang:** Resources. **Xiaoyun He:** Resources. **Yanyan Zhu:** Resources. **Yuanyuan Wang:** Project administration; Supervision; Writing‐review & editing. **Ming‐Xing Chu:** Conceptualization; Funding acquisition; Project administration; Supervision; Writing‐review & editing.

## ETHICAL STATEMENT

All the experimental procedures mentioned in the present study were approved by the Science Research Department (in charge of animal welfare issues) of the Institute of Animal Sciences, Chinese Academy of Agricultural Sciences (IAS‐CAAS) (Beijing, China). Ethical approval on animal survival was given by the animal ethics committee of IAS‐CAAS (No. IAS2020‐63).

### PEER REVIEW

The peer review history for this article is available at https://publons.com/publon/10.1002/vms3.485.
